# Chemically Diverse Toxicants Converge on Fyn and c-Cbl to Disrupt Precursor Cell Function 

**DOI:** 10.1371/journal.pbio.0050035

**Published:** 2007-02-06

**Authors:** Zaibo Li, Tiefei Dong, Chris Pröschel, Mark Noble

**Affiliations:** Department of Biomedical Genetics, University of Rochester Medical Center, Rochester, New York, United States of America; Albany Medical College, United States of America

## Abstract

Identification of common mechanistic principles that shed light on the action of the many chemically diverse toxicants to which we are exposed is of central importance in understanding how toxicants disrupt normal cellular function and in developing more effective means of protecting against such effects. Of particular importance is identifying mechanisms operative at environmentally relevant toxicant exposure levels. Chemically diverse toxicants exhibit striking convergence, at environmentally relevant exposure levels, on pathway-specific disruption of receptor tyrosine kinase (RTK) signaling required for cell division in central nervous system (CNS) progenitor cells. Relatively small toxicant-induced increases in oxidative status are associated with Fyn kinase activation, leading to secondary activation of the c-Cbl ubiquitin ligase. Fyn/c-Cbl pathway activation by these pro-oxidative changes causes specific reductions, in vitro and in vivo, in levels of the c-Cbl target platelet-derived growth factor receptor-α and other c-Cbl targets, but not of the TrkC RTK (which is not a c-Cbl target). Sequential Fyn and c-Cbl activation, with consequent pathway-specific suppression of RTK signaling, is induced by levels of methylmercury and lead that affect large segments of the population, as well as by paraquat, an organic herbicide. Our results identify a novel regulatory pathway of oxidant-mediated Fyn/c-Cbl activation as a shared mechanism of action of chemically diverse toxicants at environmentally relevant levels, and as a means by which increased oxidative status may disrupt mitogenic signaling. These results provide one of a small number of general mechanistic principles in toxicology, and the only such principle integrating toxicology, precursor cell biology, redox biology, and signaling pathway analysis in a predictive framework of broad potential relevance to the understanding of pro-oxidant–mediated disruption of normal development.

## Abstract 2

Chemically different toxins (lead, methylmercury, and paraquat) each cause the intracellular environment to become more oxidized, and thereby activate a common pathway that suppresses signaling from growth factor receptors that may be associated with developmental impairments.

## Author Summary

Discovering general principles underlying the effects of toxicant exposure on biological systems is one of the central challenges of toxicological research. We have discovered a previously unrecognized regulatory pathway on which chemically diverse toxicants converge, at environmentally relevant exposure levels, to disrupt the function of progenitor cells of the developing central nervous system. We found that the ability of low levels of methylmercury, lead, and paraquat to make progenitor cells more oxidized causes activation of an enzyme called Fyn kinase. Activated Fyn then activates another enzyme (c-Cbl) that modifies specific proteins—receptors that are required for cell division and survival—to initiate the proteins' degradation. By enhancing degradation of these receptors, their downstream signaling functions are repressed. Analysis of developmental exposure to methylmercury provided evidence that this same pathway is activated in vivo by environmentally relevant toxicant levels. The remarkable sensitivity of progenitor cells to low levels of toxicant exposure, and the discovery of the redox/Fyn/c-Cbl pathway as a mechanism by which small increases in oxidative status can markedly alter cell function, provide a novel and specific means by which exposure to chemically diverse toxicants might perturb normal development. In addition, the principles revealed in our studies appear likely to have broad applicability in understanding the regulation of cell function by alterations in redox balance, regardless of how they might be generated.

## Introduction

Determining whether chemically diverse substances induce similar adverse effects at the cellular and molecular level is one of the central challenges of toxicological research. If the structural diversity of different toxicants, and of potential toxicants, means that each works through distinctive mechanisms then this creates a potentially unsolvable challenge in developing means of screening the many tens of thousands of different chemicals for which little or no toxicological information exists. In contrast, the identification of general principles that transcend the specific chemistries of individual substances has the potential of providing broadly relevant insights into the means by which toxicants disrupt normal development. If such principles were found to apply to the analysis of toxicant levels frequently encountered in the environment, this would be of even greater potential importance in providing efficient means of analyzing this diverse array of chemicals.

Of all of the effects associated with toxicant exposure, one of the few that appears to be common to multiple chemically diverse substances is the ability of these agents to cause cells to become more oxidized. The range of toxicants reported to alter oxidative status is very broad, and includes metal toxicants such as methylmercury (MeHg; e.g., [1–6], lead [Pb] [6–9], and organotin compounds [1,2,5,10,11]), cadmium [12,13], and arsenic [12,14]. Ethanol exposure also is associated with oxidative stress [15], as is exposure to a diverse assortment of agricultural chemicals [16], including herbicides (e.g., paraquat [17,18]), pyrethroids [19–21], and organophosphate and carbamate inhibitors of cholinesterase [22–26]). Thus, the ability to cause cells to become more oxidized is shared by many toxicants, regardless of their chemical structure.

The observations that chemically diverse toxicants share the property of making cells more oxidized is of particular interest in light of the increasing evidence that oxidative regulation is a central modulator of normal physiological function. Although increases in oxidative status in a cell have been most extensively studied in the context of their adverse effects (in particular, the induction of cell death or of cell senescence), multiple studies have demonstrated that changes in redox state as small as 15%–20% may be critical in regulating such normal cellular processes as signal transduction, division, differentiation, and transcription (reviewed in, e.g., [27–31]. Although the mechanistic basis for such regulation is frequently unclear, the importance of redox status in modulating cell function makes convergence of different toxicants on this physiological parameter a matter of considerable potential interest.

Despite the observations that many toxicants share the property of making cells more oxidized, multiple questions exist regarding the relevance of such observations for the understanding of toxicant function.

First, there is considerable uncertainty about the relative importance of effects on redox state in the analysis of individual toxicants, and it is generally believed that the major effects of toxicants on cellular function are distinct from any effects on oxidative status. For example, in the context of agents analyzed in the present studies, MeHg-mediated effects on cellular function generally are thought to be mediated through binding to cysteine residues, thus disrupting function of microtubules and other proteins, but may also involve disruption of Ca^2+^ homeostasis (e.g., [32–34]). In contrast, Pb does not bind to cysteine residues and instead is thought to exert its functions through altering normal calcium metabolism by mimicking calcium action and/or by disrupting calcium homeostasis (e.g., [35,36]). This would lead to alterations in function of multiple proteins, of which the most extensively studied have been members of the protein kinase C (PKC) family of enzymes (e.g., [37,38]).

A further concern is the general lack of knowledge about whether, or where, oxidation induced by different means would mechanistically converge. For example, MeHg has been suggested to cause oxidative stress by a variety of mechanisms, including by binding to thiols, by causing a depletion in glutathione levels, or by impairing mitochondrial function [39,40], whereas Pb is thought to disrupt mitochondrial function through its effects on calcium metabolism (e.g., [35,36,41–45]) The organic herbicide paraquat (the third agent examined in the present studies) is another example of a toxicant with pro-oxidant activities, but in this case, resulting from initiation of a cyclic oxidation/reduction process in which paraquat first undergoes one electron reduction by NADPH to form free radicals that donate their electron to O_2_, producing a superoxide radical; upon exhaustion of NADPH, superoxide reacts with itself and produces hydroxyl free radicals (e.g., [17,18]). Whether these different means of altering oxidative state would have different mechanistic consequences is unknown.

A further concern regarding the hypothesis that changes in redox state represent an important convergence point of toxicant action is whether oxidative changes are even associated with toxicant exposure at levels frequently encountered in the environment. For example, although several studies have documented the ability of MeHg to cause cells to become more oxidized, effective exposure levels employed in these studies have generally ranged from 1–20 μM [2–5], which is 30–600 times the upper range of average mercury concentrations found in the bloodstream of as many as 600,000 newborn infants in the United States alone [46]. Similar concerns apply to the analysis of multiple toxicants, for which pro-oxidant effects have largely been studied at exposure levels much higher than those with broad environmental relevance.

In addition, a more general concern regarding the search for general principles of toxicant action is whether such convergence, if it exists, would occur only at exposure levels that induce cell death or whether common mechanisms might be relevant to the understanding of more subtle effects of toxicant exposure, particularly during critical developmental periods. Because development is a cumulative process, the effects of small changes in, e.g., progenitor cell division and/or differentiation, that are maintained over multiple cellular generations could have substantial effects on the organism. Such changes are poorly understood, however, at both cellular and molecular levels.

Our present studies have led to the discovery of a previously unrecognized regulatory pathway on which environmentally relevant levels of chemically diverse toxicants converge to compromise division of a progenitor cell isolated from the developing central nervous system (CNS). We found that exposure of cells to low levels of MeHg, Pb, or paraquat is sufficient to make cells more oxidized and to activate Fyn kinase, a Src family member known to be activated by increased oxidative status. This first step activates a pathway wherein Fyn activates c-Cbl, a ubiquitin ligase that plays a critical role in modulating degradation of a specific subset of receptor tyrosine kinases (RTKs). c-Cbl activation in turn leads to reductions in levels of target RTKs, thus suppressing division of glial progenitor cells. The effects of all three toxicants are blocked by co-exposure to N-acetyl-L-cysteine, which is widely used to protect against oxidative stress. We also provide evidence that our in vitro analyses successfully predict previously unrecognized effects of developmental MeHg exposure at levels 90% below those previously considered to represent low-dose exposure levels.

## Results

### Exposure to Environmentally Relevant Levels of MeHg Causes Glial Progenitor Cells to Become More Oxidized and Suppresses Their Division

The progenitor cells that give rise to the myelin-forming oligodendrocytes of the CNS offer multiple unique advantages for the study of toxicant action, particularly in the context of analysis of toxicant effects mediated by changes in intracellular redox state. These progenitors (which are referred to as both oligodendrocyte-type-2 astrocyte [O-2A] progenitor cells ([47] and oligodendrocyte precursor cells, here abbreviated as O-2A/OPCs) are one of the most extensively studied of progenitor cell populations (reviewed in, e.g., [48–52]. They also are among a small number of primary cell types that can be analyzed as purified populations, and at the clonal level, and for which there is both extensive information on the regulation of their development and also evidence of their importance as targets of multiple toxicants (including such chemically diverse substances as Pb [38,53], ethanol [e.g., [54–57]], and triethyltin [10,58]).

**Figure 1:**
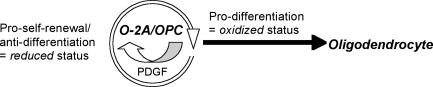
Diagrammatic Summary of the Role of Redox Regulation in Modulating Division and Differentiation of O-2A/OPCs Progenitor cells are induced to divide by exposure to PDGF. Induced to divide by PDGF alone, progenitors will undergo a limited number of divisions while asymmetrically generating oligodendrocytes. The balance between division and differentiation is modulated, however, by the intracellular redox state [59]. Cells that are more oxidized tend to differentiate, whereas those that are more reduced undergo more self-renewal. Pharmacological agents that make cells more oxidized induce differentiation of dividing O-2A/OPCs into oligodendrocytes. Similarly, signaling molecules that induce differentiation (e.g, TH) make cells more oxidized as a necessary part of their mechanism of action. In contrast, pharmacological agents that make cells more reduced promote self-renewal, and signaling molecules that enhance self-renewal (e.g, NT-3) make cells more reduced as a necessary part of their mechanism of action.

Another important feature of O-2A/OPCs, in regard to the present studies, is that their responsiveness to small (∼15%–20%) changes in the intracellular redox state provides a central integrating mechanism for the control of their division and differentiation [59]. O-2A/OPCs purified from developing animals on the basis of the cell's intracellular redox state exhibit strikingly different propensities to divide or differentiate. Cells that are more reduced at the time of their isolation undergo extended division when grown in the presence of platelet-derived growth factor (PDGF, the major mitogen for O-2A/OPCs [60–62]), whereas those that are more oxidized are more prone to undergo differentiation [59]. Pharmacological agents that make cells slightly more reduced enhance self-renewal of dividing progenitors, whereas pharmacological agents that make cells more oxidized, by as little as 15%–20%, suppress division and induce oligodendrocyte generation. Moreover, cell-extrinsic signaling molecules (e.g., neurotrophin-3 [NT-3] and fibroblast growth factor-2 [FGF-2]) that enhance the self-renewal of progenitors dividing in response to PDGF cause cells to become more reduced. In contrast, signaling molecules that induce differentiation to oligodendrocytes (i.e., thyroid hormone [TH] [63,64]) or astrocytes (i.e., bone morphogenetic protein-4 [BMP-4] [65,66]) cause cells to become more oxidized [59]. The ability of these signaling molecules to alter redox state is essential to their mechanisms of action, because pharmacological inhibition of the redox changes they induce blocks their effects on either division or differentiation of O-2A/OPCs. Thus, multiple lines of evidence have demonstrated that responsiveness to small changes in redox status represents a central physiological control point in these progenitor cells (as summarized in Figure 1).

We initiated our studies of toxicant effects on O-2A/OPCs with an examination of MeHg, which has been previously studied for its effects on neuronal migration, differentiation, and survival, and on astrocyte function (e.g., [67–74]). Little is known about the effects of MeHg on the oligodendrocyte lineage, despite the fact that there are several reports over the past two decades documenting decreases in conduction velocity in the auditory brainstem response (ABR) of MeHg-exposed children [75–78] and rats [79]. Such a physiological alteration has long been considered to be indicative of myelination abnormalities in children whose development has been compromised by iron deficiency (see, e.g., [80,81]).

**Figure 2:**
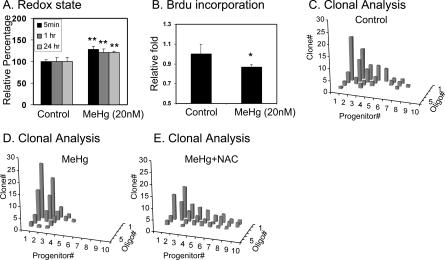
MeHg Exposure Makes O-2A/OPCs More Oxidized, Suppresses BrdU Incorporation, and Decreases Cell Division in Clonal Assays (A) Purified O-2A/OPCs were grown in the presence of 10-ng/ml PDGF overnight. Effects of MeHg on intracellular redox state were determined by analysis of 2′,7′-dichlorodihydrofluorescein diacetate fluorescence emission in O-2A progenitors exposed to 20 nM MeHg for various lengths of time, as indicated. (B) Cells were plated in medium containing PDGF and then exposed to 20 nM MeHg for an additional 72 h. During the last 4 h of exposure, cultures were also exposed to BrdU. Cultures were then stained with A2B5 and anti-BrdU antibodies (to recognize all progenitors and those synthesizing DNA during the BrdU pulse, respectively). Results are presented as comparison with control cultures. (C–E) Suppression of cell division by exposure to MeHg was studied in more detail at the clonal level. Cells were treated as for (B), except for being plated at clonal density (as in [59,199]). Cultures were maintained for 6 d, and then 100 randomly chosen clones were analyzed for their composition (as in [59,64,199]). Data are presented, for all clones analyzed, in three dimensions such that the *x*-axis equals the number of progenitors per clone, the *z*-axis equals the number of oligodendrocytes (Oligo) per clone, and the *y*-axis equals the number of clones with any given composition. In cultures exposed to MeHg, there was a decrease in the representation of large clones and a proportionate increase in the number of small clones and clones containing oligodendrocytes. The effects of MeHg were prevented by co-exposure of cells to 1 mM N-acetyl-L-cysteine (NAC). All experiments were repeated at least three times, and all numerical values represent means ± SD for triplicate data points.

We found that exposure of O-2A/OPCs (growing in chemically defined medium supplemented with PDGF) to environmentally relevant levels of MeHg makes these cells approximately 20% more oxidized (Figure 2A), a degree of change similar to that previously associated with reductions in progenitor cell division [59]. Exposure to MeHg inhibited progenitor cell division as determined both by analysis of bromodeoxyuridine (BrdU) incorporation (Figure 2B) and by analysis of cell division in individual clones of O-2A/OPCs (Figure 2C–2E). These oxidizing effects of MeHg were seen at exposure levels as low as 20 nM, less than the 5.8 μg/l or more (i.e., parts per billion [ppb]) of MeHg found in cord blood specimens of as many as 600,000 infants in the US each year [46] and 0.3% or less of the exposure levels previously found to induce oxidative changes in astrocytes [4]. Exposure to 20 nM MeHg was sufficient to cause an approximately 25% drop in the percentage of O-2A/OPCs incorporating BrdU in response to stimulation with PDGF. When examined at the clonal level, MeHg exposure was associated with a reduction in the number of large clones and an increase in the number of small clones, as seen for other pro-oxidant stimuli [59]. Increasing MeHg exposure levels above 50 nM was associated with significant lethality, but little or no cell death was observed at the lower concentrations used in the present studies (unpublished data). Thus, division of O-2A/OPCs exhibits a striking sensitivity to low concentrations of MeHg.

### MeHg Exposure Reduces the Effects of PDGF from the Nucleus Back to the Receptor

**Figure 3:**
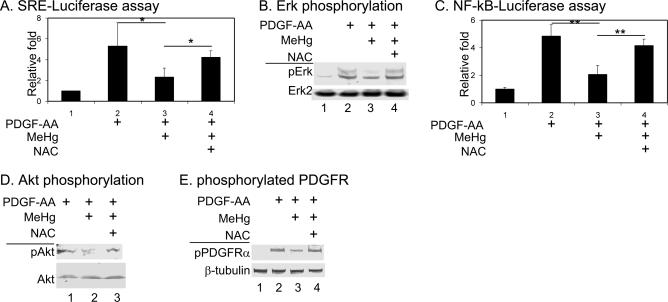
MeHg Suppresses PDGF-Mediated Signaling from the Nucleus Back to the Receptor (A) Progenitors transfected with an SRE-luciferase reporter construct and exposed to 30 nM MeHg (24 h) showed significantly lower levels of reporter activity. *, *p* < 0.05. (B) Cells grown as in (A) and analyzed for phosphorylation of ERK1/2 showed reduced ERK1/2 phosphorylation. (C) Cells transfected with an Nf-κB-luciferase reporter construct and treated as in (A) showed reduced Nf-κB transcriptional activity. **, *p* < 0.01. (D) Cells grown as in (A) showed lower levels of Akt (Thr 308) phosphorylation. (E) Cells grown as in (A) also showed decreased phosphorylation of PDGFRα (as detected with anti-PDGFRα(pY^742^) antibody). All effects of MeHg were prevented by growth of cells in the additional presence of 1 mM NAC. All experiments were repeated at least three times, and all numerical values represent means ± SD for triplicate data points. The plus symbol indicates exposure of the cells to the indicated substance.

One possible explanation for the reduced division associated with MeHg exposure would be disruption of PDGF-mediated signaling, and molecular analysis revealed that exposure of O-2A/OPCs to 30 nM MeHg for 24 h suppressed PDGF-induced signaling pathway activation at multiple points from the nucleus back to the receptor. One pathway stimulated by PDGF binding to the PDGF receptor-α (PDGFRα) leads to sequential activation of Raf-1, Raf-kinase, and extracellular signal-regulated kinase 1 and 2 (ERK1/2), which further leads to activation of the Elk-1 transcription factor and up-regulation of immediate early-response gene expression, at least in part through activation of the serum response element (SRE) promoter sequence [82,83]. MeHg exposure was associated with reduced expression of an SRE-luciferase reporter gene (Figure 3A), and reduced ERK1/2 phosphorylation (Figure 3B). PDGFRα activation also stimulates activity of PI-3 kinase, leading to activation of Akt and induction of NF-κB–mediated transcription (e.g., [82,84,85]), both of which also were inhibited by MeHg exposure. Expression of an NF-κB-luciferase reporter gene was decreased (Figure 3C), as was phosphorylation of Akt (Figure 3D). Phosphorylation of PDGFRα, indicating receptor activation, was also reduced in cells exposed to MeHg (Figure 3E). Because O-2A/OPCs growing in these cultures are absolutely dependent upon PDGF for continued division (e.g., [60,61,86]), the suppression of PDGF signaling would necessarily cause a reduction in cell division.

### Pathway-Specific Disruption of PDGF-Mediated Signaling, and Reductions in Levels of PDGFRα, Induced by MeHg

**Figure 4:**
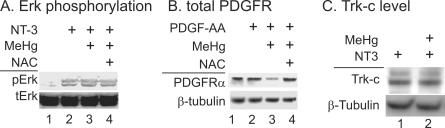
MeHg Effects Are Pathway Specific and Are Associated with Reduced Levels of PDGFRα (A) MeHg does not inhibit ERK1/2 phosphorylation induced by exposure to NT-3. Cells were grown as in Figure 2A, but exposed to NT-3 instead of PDGF. As shown, MeHg exposure did not reduce the extent of ERK1/2 phosphorylation induced by exposure to NT-3, thus indicating that the site of action of MeHg is not on the level of these kinases. (B) Consistent with the indication from these results that effects of MeHg were mediated further upstream in the PDGF-signaling pathway, analysis of cells treated in the same manner showed reductions in total levels of PDGFRα. The effects of MeHg on total levels of PDGFRα were prevented by co-exposure with NAC. (C) Exposure to MeHg was not associated with reductions in levels of TrkC, indicating that receptor loss was mediated by a mechanism that distinguishes between PDGFRα and TrkC. All experiments were repeated at least three times. The plus symbol indicates exposure of the cells to the indicated substance.

We next found that the effects of MeHg were pathway specific and were associated with reductions in total levels of PDGFRα. O-2A/OPCs exposed to 30 nM MeHg exhibited no reduction in ERK1/2 phosphorylation induced by exposure to NT-3 (Figure 4A), and no reduction in NT-3–induced expression from an SRE-luciferase reporter construct (unpublished data). This result suggested that the site of action of MeHg was upstream of ERK1/2 regulation, prompting us to look directly at the PDGFRα. We found that the reduction in phosphorylated PDGFRα (Figure 3E) was paralleled by a reduction in levels of the PDGFRα itself (Figure 4B). In contrast, no reduction in levels of TrkC (the receptor for NT-3 [87]) was caused by exposure to MeHg (Figure 4C).

### Fyn and c-Cbl Activation, and Enhanced Degradation of PDGFRα Induced by MeHg

One possible explanation for the ability of MeHg to cause a reduction in PDGF-mediated signaling and in total levels of PDGFRα, without affecting NT-3–mediated signaling or TrkC levels, would be that exposure to this toxicant leads to activation of c-Cbl, an E3 ubiquitin ligase that ubiquitylates the activated PDGFRα [88,89], thus leading to its internalization and potential lysosomal degradation [90–92]. Such a possibility is particularly intriguing in light of multiple reports that c-Cbl can be activated by Fyn kinase (e.g., [93–96]), a Src family kinase that can be activated by oxidative stress [97–100]. O-2A/OPCs are known to express Fyn, which has been studied in these cells for its effects on regulation of RhoA activity and control of cytoskeletal organization [101,102]. Because TrkC does not appear to be regulated by c-Cbl, redox-modulated activation of Fyn, leading to c-Cbl activation and enhanced PDGFRα degradation, would provide a potential mechanistic explanation integrating the observations reported thus far.

**Figure 5:**
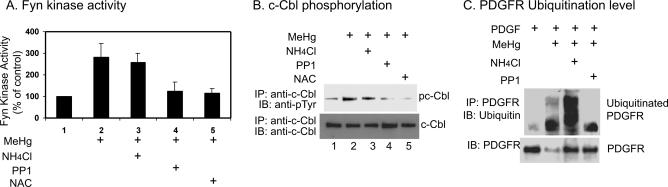
MeHg Exposure Causes Activation of Fyn, Phosphorylation of c-Cbl, and Ubiquitylation of PDGFRα Leading to Reductions in Receptor Level (A) O-2A/OPCs exposed to 30 nM MeHg exhibited higher levels of Fyn kinase activity, as detected by analysis of immunoprecipitated Fyn from these cells using the Universal Tyrosine Kinase Assay Kit (Takara), as described in Materials and Methods. Values (mean ± SD) are expressed as the percent of controls, which were defined from basal Fyn kinase activity without any stimulation. All bars with increased levels of Fyn activity differ from control values at *p* < 0.001. Increased Fyn activity was blocked by pre-treatment of cells with NAC or with the src-family kinase inhibitor PP1, but not by pre-treatment with NH_4_Cl (an inhibitor of lysosomal function). (B) As for (A), Increased c-Cbl phosphorylation (see Materials and Methods for immunoprecipitation assay) associated with MeHg exposure was blocked by co-exposure of cells to NAC or PP1, but was not blocked when cells were exposed to NH_4_Cl. (C) Exposure to MeHg was associated with a marked increase in the levels of ubiquitylation of PDGFRα, with this increase being apparent even though receptor levels were themselves reduced by toxicant exposure. NH_4_Cl treatment was associated with rescue of levels of total PDGFRα, and therefore was associated with a still more marked increase in the amount of ubquitylated receptor detected. As predicted by the hypothesis that both receptor ubiquitylation and receptor loss are due to activation of Fyn, co-exposure of cells to PP1 rescued receptor levels and greatly reduced the extent of receptor ubiquitylation. The plus symbol (+) indicates exposure of the cells to the indicated substance. IB = immunoblot; IP = immunoprecipitation.

A variety of data support the hypothesis that MeHg exposure activates Fyn, leading to activation of c-Cbl, followed by degradation-mediated reductions in levels of activated PDGFRα. Exposure of O-2A/OPCs to 30 nM MeHg stimulated Fyn activation and c-Cbl phosphorylation (Figure 5A and 5B). Activation of Fyn and c-Cbl was blocked by the Src family kinase inhibitors PP1 (Figure 5A and 5B) and PP2 (unpublished data). We next found that exposure to MeHg enhanced ubiquitylation of PDGFRα (a predicted consequence of c-Cbl activation), an increase readily observed even in the presence of markedly reduced levels of the receptor itself (Figure 5C). Co-exposure to ammonium chloride (NH_4_Cl, a lysosomotropic weak base that increases lysosomal pH and disrupts lysosomal protein degradation [103–105]) prevented receptor degradation, and was associated with increased levels of ubiquitylated receptor in treated O-2A/OPCs. The increase in levels of ubiquitylated receptor was as predicted by the lack of effect of NH_4_Cl on either Fyn activation or c-Cbl phosphorylation (Figure 5A and 5B). Treatment with PP1, which inhibits Fyn activity (Figure 5A), was also associated with a marked reduction in the amount of ubiquitylated PDGFRα, particularly in comparison with levels of total receptor (compare upper and lower lanes in Figure 5C). As further confirmation that reductions in levels of PDGFRα were due to protein degradation, exposure to MeHg did not have any significant effects on levels of PDGFRα mRNA, as determined by quantitative PCR analysis (Figure S1A). In the presence of cycloheximide, an inhibitor of protein synthesis, MeHg further accelerated receptor loss as compared with that occurring solely due to failure to synthesize new protein (Figure S1B). Collectively, these results indicate that MeHg enhances active degradation of PDGFRα, as contrasted with reducing receptor levels as an indirect consequence of altering transcriptional or translational regulation of receptor levels.

**Figure 6:**
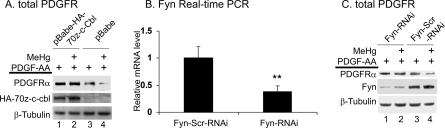
MeHg-Induced Reductions in PDGFRα Levels Were Prevented by Expression of DN(70Z) c-Cbl and by Expression of Fyn-Specific RNAi Constructs (A) Expression (by transfection, as described in Materials and Methods) of DN(70Z) c-Cbl prevented MeHg-induced reductions in levels of PDGFRα, an effect not obtained with vector alone (pBabe). (B) Expression of Fyn-specific RNAi (as described in Materials and Methods) caused a reduction in levels of Fyn protein, whereas scrambled (Scr) controls had no effect on levels of this protein. Data are presented as comparisons with levels of Fyn protein in non-manipulated cells. (C) Expression of Fyn-RNAi constructs, but not of Fyn-Scr-RNAi protected O-2A/OPCs from MeHg-induced reductions in levels of total PDGFRα. Fyn RNAi constructs had no effects on levels of tubulin or on levels of c-Cbl (unpublished data). All experiments were repeated at least three times. The plus symbol indicates exposure of the cells to the indicated substance.

Molecular confirmation of the role of Fyn and c-Cbl in the effects of MeHg on levels of PDGFRα was obtained by expression of dominant negative c-Cbl, or small inhibitory RNA (RNAi) for Fyn or Cbl, in MeHg-exposed O-2A/OPCs. Expression of the dominant-negative (DN) 70z mutant of c-Cbl [106–108] in O-2A/OPCs prevented MeHg-induced reductions in levels of PDGFRα (Figure 6A). Reduction in levels of Fyn protein by introduction of Fyn-specific small interfering RNA (siRNA) constructs (Figure 6B) also protected against MeHg-induced reductions in levels of PDGFRα (Figure 6C), as predicted by the hypothesis that MeHg-induced activation of Fyn mechanistically precedes reductions in receptor levels. Similar results were obtained using RNAi constructs for c-Cbl, but are presented later in the paper, in the context of analysis of other toxicants.

**Figure 7:**
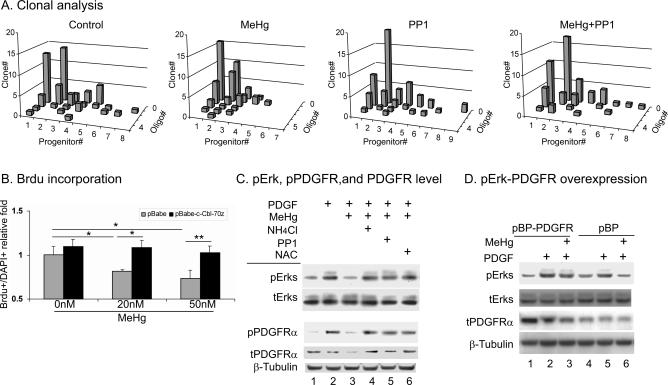
Inhibition of Fyn Activity, c-Cbl Activity, or of Lysosomal Function Rescues Cell Division and/or ERK1/2 Phosphorylation in MeHg-Treated O-2A/OPCs (A) Purified O-2A/OPCs were plated at clonal density and analyzed as for Figure 2. After 24 h, MeHg was added to cultures in the presence or absence of PP1. MeHg by itself was associated with a reduction in the contribution of large clones dominated by progenitors and an increased representation of smaller clones and of oligodendrocytes. Co-exposure to PP1 rescued cells from the effects of MeHg. (B) Expression of DN(70Z) c-Cbl rescued purified progenitors from MeHg-associated suppression of BrdU incorporation. It was notable that expression of DN(70Z) c-Cbl also rescued cells from the effects of 50 nM MeHg, raising the possibility that further exploration of this pathway will reveal additional roles in cell survival (a topic to be explored in future research). *, *p* < 0.05; **, *p* < 0.01. (C) Co-exposure of cells to NH_4_Cl or PP1 together with MeHg protected cells from MeHg-induced suppression of ERK1/2 and PDGFRα phosphorylation and reductions in total levels of PDGFRα. (D) Overexpression of PDGFRα also rescued cells from MeHg-induced suppression of ERK1/2 phosphorylation, whereas expression of a control construct (pBP) did not rescue ERK1/2 phosphorylation. All experiments were repeated at least three times, and all numerical values represent means ± SD for triplicate data points. The plus symbol (+) indicates exposure of the cells to the indicated substance.

Suppression of Fyn or c-Cbl activity, or overexpression of PDGFRα itself, also protected against the functional effects of MeHg exposure (Figure 7). Pharmacological inhibition of Fyn activity with PP1 enabled analysis of O-2A/OPC division at the clonal level, and demonstrated that PP1 blocked MeHg-induced suppression of cell division (Figure 7A). O-2A/OPCs expressing DN-70Z-c-Cbl and exposed to MeHg were also protected from effects of MeHg on cell division, as analyzed by BrdU incorporation (Figure 7B). Co-treatment of MeHg-exposed O-2A/OPCs with PP1 or NH_4_Cl also blocked MeHg-associated suppression of ERK1/2 phosphorylation (and MeHg-induced reductions in levels of PDGFRα, indicating that ERK1/2 suppression was a secondary consequence of the effects of Fyn and c-Cbl activation (Figure 7C). Overexpression of PDGFRα in MeHg-exposed O-2A/OPCs also protected cells from MeHg-associated reductions in ERK1/2 phosphorylation (Figure 7D).

### Convergence of Chemically Diverse Toxicants on Activation of Fyn and c-Cbl, and Reductions in Levels of PDGFRα

To determine whether effects of MeHg revealed a general mechanism by which chemically diverse toxicants with pro-oxidant activity could alter cellular function in similar ways, we next examined the effects of exposure of dividing O-2A/OPCs to Pb (a heavy metal toxicant) and paraquat (an organic herbicide). As discussed in the Introduction, these toxicants both make cells more oxidized, but through mechanisms that differ between them and also from effects of MeHg.

**Figure 8:**
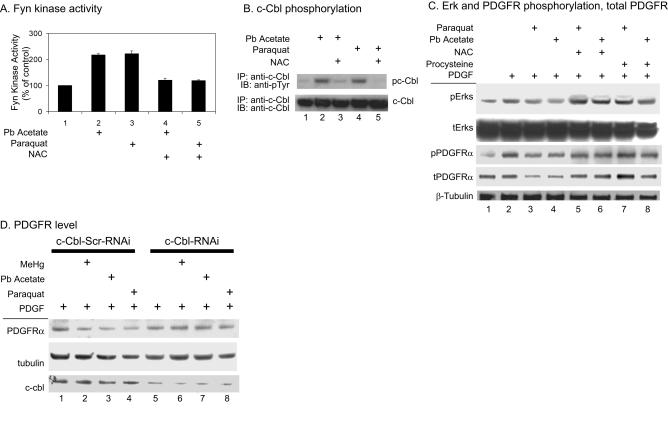
Pb and Paraquat Exposure Caused Activation of Fyn and c-Cbl, Suppression of ERK1/2 Phosphorylation, and Reduction in Levels of PDGFRα (A) Purified O-2A/OPCs were treated as for analysis of MeHg, except that cells were exposed to 1 μM Pb or 5 μM paraquat. Both toxicants caused activation of Fyn, analyzed as in Figure 5. (B) Pb and paraquat exposure also caused phosphorylation of c-Cbl, as detected by immunoprecipitation of total c-Cbl followed by analysis with anti-phosphotyrosine antibody. (C) Pb and paraquat exposure cause suppression of ERK1/2 phosphorylation and reductions in total levels of PDGFRα. (D) Expression of c-Cbl RNAi caused a reduction in levels of c-Cbl protein and protected PDGFRα levels from effects of MeHg, Pb, and paraquat, whereas scrambled (Scr) RNAi constructs had no levels of PDGFRα. NAC (or procysteine, a cysteine pro-drug with no intrinsic anti-oxidant activity) protected against all effects of toxicant exposure. All experiments were repeated at least three times. The plus symbol indicates exposure of the cells to the indicated substance.

Despite their chemical differences from MeHg, and from each other, Pb and paraquat had apparently identical effects as MeHg on ERK1/2 phosphorylation, activation of Fyn and c-Cbl, and reductions in levels of phosphorylated PDGFRα and on total levels of PDGFRα (Figure 8). O-2A/OPCs were exposed to 1 μM Pb (equivalent to the level of 20 μg/dl that is known to be associated with cognitive impairment, and a level of Pb previously found to inhibit O2A/OPC division without causing cell death [38,53,109]) or to 5 μM paraquat (an exposure level selected as being in the lowest 0.1% of the range of paraquat concentrations studied by others in vitro, which range from 8 μM–300 mM (e.g., [110–114]). Pb and paraquat exposure at these levels did not cause cell death, but did make O-2A/OPCs approximately 20% more oxidized, as determined by analysis of cells with the redox-indicator dyes dihydro-chloromethyl-rosamine or dihydro-calcein-AM (unpublished data). Both Pb and paraquat exposure were associated with activation of Fyn (Figure 8A), increased phosphorylation of c-Cbl (Figure 8B), reduced levels of ERK1/2 phosphorylation, and reduced levels of phosphorylated and total PDGFRα (Figure 8C). As for MeHg, the effects of Pb and paraquat on PDGFRα levels were prevented by expression of RNAi for c-Cbl (Figure 8D), DN(70Z) c-Cbl, or RNAi for Fyn (unpublished data).

It has previously been suggested that the effects of Pb on O-2A/OPCs are mediated through activation of PKC [38], a pathway that has not been implicated in the activity of MeHg or paraquat. To determine whether PKC inhibition could distinguish between effects of Pb versus MeHg or paraquat, and to determine if PKC activation was relevant to the effects of toxicants on Fyn or c-Cbl activation or reductions in PDGFRα levels, we next examined the effects of co-exposure of O-2A/OPCs to bisindolylmaleimide I (BIM-1, a broad-spectrum PKC inhibitor previously used in the analysis of the role of PKC activation in the effects of Pb on O-2A/OPCs [38]). As shown in Figure S2, we found that co-exposure of O-2A/OPCs to BIM-1 with Pb, MeHg, or paraquat did not prevent toxicant-mediated activation of Fyn (Figure S2A) or c-Cbl (Figure S2B). BIM-1 co-exposure also did not protect against MeHg-, Pb- or paraquat-induced reductions in levels of PDGFRα (Figure S2C).

### Protection by Cysteine Pro-Drugs

If it is correct that Fyn activation, with its consequences, is regulated by the ability of toxicants to make cells more oxidized, then antagonizing such redox changes should prevent Fyn activation. Previous studies have shown that an effective means of preventing the increase in oxidative status and the suppression of cell division caused by exposure of O-2A/OPCs to TH is to treat cells with N-acetyl-L-cysteine (NAC), a cysteine pro-drug that is readily taken up by cells and converted to cysteine [59]. Cysteine is the rate-limiting precursor for synthesis of glutathione, one of the major regulators of intracellular redox status (e.g., [115,116]. NAC also possesses anti-oxidant activity, has long been used as a protector against many types of oxidative stress (e.g., [9,117,118]), and has been shown to confer protection against a wide range of toxicants, including MeHg (e.g., [119–121]), Pb (e.g., [9,122,123]), and paraquat (e.g., [17,124]), as well as such other substances as aluminum [125], cadmium [126], arsenic [127], and cocaine [128].

As predicted by the hypothesis that the pro-oxidant activities of chemically diverse toxicants are causal in Fyn activation, NAC was equally effective at preventing Fyn activation—and its consequences—induced by exposure to MeHg, Pb, or paraquat (Figures 2–5, 7, and 8). For cells grown at the clonal level, NAC blocked the suppressive effects of MeHg on cell division (Figure 2). NAC also blocked all effects of MeHg on PDGF-mediated signaling, and rescued normal level of activity of SRE and NF-kB promoter-reporter constructs and levels of phosphorylation of ERK1/2, Akt, and PDGFRα (Figure 3). Consistent with the hypothesis that Fyn is activated when cells become more oxidized [97–100], NAC also blocked MeHg-induced activation of Fyn and phosphorylation of c-Cbl (Figure 5), and prevented MeHg-induced reductions in levels of PDGFRα (Figure 4). Critically, for the hypothesis that Pb and paraquat effects also were mediated by changes in redox state, NAC also blocked the effects of Pb and paraquat on Fyn activation and c-Cbl phosphorylation, and protected against effects of these toxicants on ERK1/2 phosphorylation and levels of PDGFR (Figure 8). Levels of PDGFRα were also protected by exposure of O-2A/OPCs to procysteine (Figure 8), a thiazolidine-derivative cysteine pro-drug that differs from NAC in having no intrinsic anti-oxidant activity [129]. Although it is conceivable that the ability of cysteine pro-drugs to protect against the effects of MeHg, Pb, and paraquat is due to enhanced toxicant clearance associated with elevated levels of glutathione, analysis of Pb uptake with Leadmium Green AM (a fluorescent indicator of Pb levels) showed no significant difference in Pb levels between cells exposed to Pb as compared with cells exposed to Pb and NAC (Figure S3).

**Figure 9:**
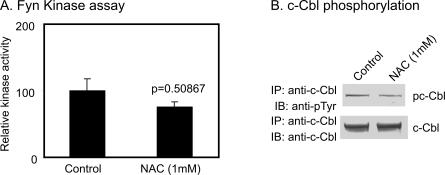
Exposure of O-2A/OPCs to 1 mM NAC Has Only Minimal Effects on Basal Activity of Fyn or Phosphorylation of c-Cbl Assays of Fyn activity and c-Cbl phosphorylation were carried out as in Figure 5, except that cells were exposed only to NAC and not to MeHg. (A) Basal Fyn activity is slightly, but not significantly, lower in cells exposed to NAC. (B) The extent of c-Cbl phosphorylation in O-2A/OPCs exposed to NAC is similar to that seen in cells grown in the presence of PDGF only. IB = immunoblot; IP = immunoprecipitation

The ability of NAC to block toxicant-induced activation of Fyn raises the question of whether this is due to a true prevention of the effects of toxicant exposure on activation of this kinase or, alternatively, is due to an ability of NAC to independently suppress Fyn activity to such an extent that the apparent block of toxicant effects instead represents the summation of two opposing influences of equivalent magnitude. To evaluate these two possibilities, O-2A/OPCs were exposed to 1 mM NAC in the absence of toxicants, and Fyn and c-Cbl activation were evaluated as in Figure 5. We found that NAC exposure had only a slight, and nonsignificant, effect on the levels of basal Fyn activity in O-2A/OPCs (Figure 9A). In agreement with this outcome, NAC exposure did not have any marked effect on levels of c-Cbl phosphorylation (Figure 9B). Thus, it appears that NAC-mediated counteraction of the effects of toxicants on Fyn activation is far greater in its magnitude than its direct effects on basal levels of Fyn activity.

### Toxicants Cause Reductions in Levels of Other c-Cbl Targets

If the hypothesis is correct that exposure of O-2A/OPCs to toxicants causes activation of the Fyn/c-Cbl pathway, then other c-Cbl targets should be affected similarly to the PDGFRα. One member of the c-Cbl interactome [92] known to be expressed by O-2A/OPCs is c-Met [130], the receptor for hepatocyte growth factor (HGF; [131,132]). Oligodendrocytes also have recently been reported to be responsive to epidermal growth factor (EGF) application with morphological changes [133], and microarray analysis confirms that the EGF receptor (EGFR) is expressed by O-2A/OPCs (C. Pröschel and M. Noble, unpublished results). The EGFR is perhaps the most extensively studied RTK target of c-Cbl [90,96,107,134–137], but c-Met regulation by c-Cbl appears to follow similar principles [106,138].

**Figure 10:**
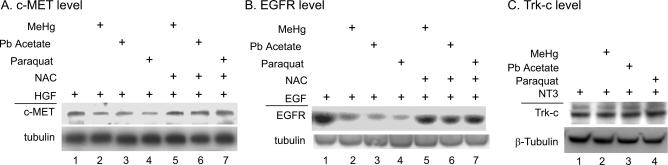
Exposure to MeHg, Pb, and Paraquat Caused Reductions in Levels of c-Cbl Targets c-Me and EGFR, but Not of TrkC Cells were analyzed as for Figure 5, but with antibodies against c-Met and EGFR. The results for the c-Cbl Targets c-Met (A), EGFR (B), and TrkC (C) are shown. All experiments were repeated at least three times.

As shown in Figure 10, exposure of O-2A/OPCs to MeHg was associated with reductions in levels of c-Met (Figure 10A) and EGFR (Figure 10B). As predicted by the hypothesis that Pb and paraquat converge with MeHg on activation of the Fyn/c-Cbl pathway, levels of C-Met and EGFR were also reduced in O-2A/OPCs exposed to these additional toxicants. Consistent with the hypothesis that such changes were associated with the ability of toxicants to make cells more oxidized, NAC protected both c-Met and EGFR levels from reductions associated with exposure to MeHg, Pb, or paraquat.

Further support for the Fyn/c-Cbl hypothesis of toxicant convergence was provided by observations that neither Pb or paraquat caused a reduction in levels of TrkC (Figure 10C), just as observed for MeHg (Figure 4C).

### Developmental Exposure to Low Levels of MeHg in Vivo Causes Reductions in Levels of PDGFRα and EGFR, but Not TrkC, and Causes Reduced Division of O-2A/OPCs

Although the central goal of the present studies was the identification of mechanistic pathways on which chemically diverse toxicants converge, it is important to also consider whether any aspects of our in vitro findings are predictive of in vivo outcomes. Although detailed in vivo investigations will be the subject of future studies, we have tested three of the key findings of our present work for which previous studies are not predictive of likely experimental outcomes.

The three questions we examined in vivo were whether toxicant exposure is associated with specific reductions in RTKs that are c-Cbl targets, whether this occurs at levels of toxicant exposure approximating the effects of environmental exposure, and whether such exposure can be shown to cause subtle changes in O-2A/OPC function. These experiments were conducted entirely with MeHg for several reasons. First, there is already extensive evidence that Pb exposure in vivo has adverse effects on myelination and on O-2A/OPCs (e.g., [38,43,53,139–142]). In contrast, evidence that MeHg exposure may have any effects on myelination thus far comes only from observations of increased latencies in ABRs [75–79], with no studies examining effects of this toxicant on the function of cells important for myelination (i.e., oligodendrocytes or their ancestral O-2A/OPCs). Third, previous studies on mice have not been conducted using levels of exposure of broad environmental relevance. Instead, such studies have defined a low exposure range as being exposure of animals to MeHg in their drinking water at a concentration of one or more parts per million (e.g., [143–147]), an exposure level considerably higher than what our studies would predict as being necessary to affect progenitor cells of the developing CNS. Thus, the question of whether MeHg exposure levels of broader environmental relevance would have any effects at all in vivo appears to be largely unaddressed.

To test the hypothesis that environmentally relevant levels of MeHg exposure can perturb the developing CNS in subtle ways, we exposed SJL mice to 100 or 250 ppb MeHg in their drinking water throughout gestation, and maintained this exposure until sacrifice of pups at 7 and 21 d after birth. As discussed in Materials and Methods, these exposure levels enabled us to approximate the predicted mercury levels in the CNS of 300,000–600,000 infants in the US. The exposure levels examined in our studies are 75%–90% below what has otherwise been considered to be low-dose exposure in mice.

**Figure 11:**
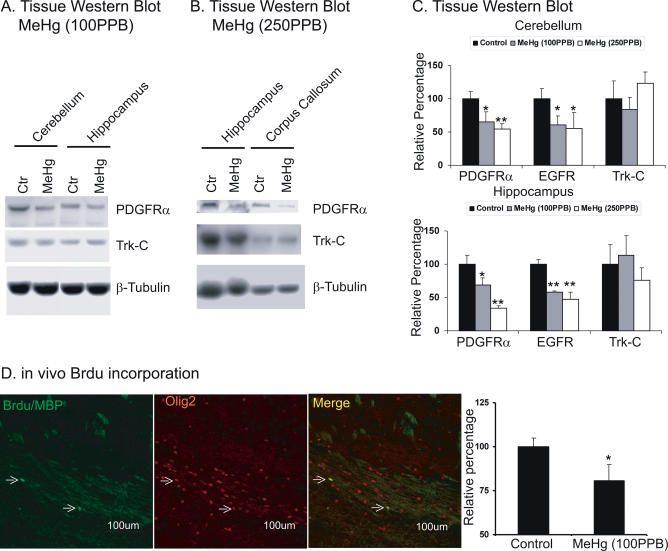
In Vivo Analysis Confirms That Developmental Exposure to Low Levels of MeHg Is Associated with Specific Reductions in Levels of PDGFRα and EGFR, but Not of TrkC, and with Reductions in O-2A/OPC Division Treatment of SJL mice with 100 or 250 ppb MeHg in the maternal drinking water during gestation and suckling was associated with reductions in levels of PDGFRα in the cerebellum and hippocampus at postnatal day 7 (P7), and in hippocampus and corpus callosum at P21. In contrast, levels of the NT-3 receptor TrkC (which does not appear to be a c-Cbl target) were not reduced in these animals. (A) Animals were treated with 100 ppb MeHg in the drinking water during pregnancy. Pups were sacrificed at 7 d after birth. Analysis of cerebellum and hippocampus showed clear reductions in levels of PDGFRα, but not in TrkC. (B) At P21, enough tissue could also be isolated from corpus callosum for analysis, and was found to have marked reductions in levels of PDGFRα, but not TrkC. (C) Quantitative analysis of receptor levels in P7 mice showed reductions in levels of PDGFRα and EGFR, but not TrkC. Quantitative analysis of changes in receptor expression in tissue from P7 mice. *, *p* < 0.05; **, *p* < 0.01) (D) Analysis of BrdU incorporation in Olig2^+^ cells reveals a reduction of approximately 20% in the number of double-positive cells in P14 animals born to mothers receiving 100 ppb MeHg in their drinking water beginning 30 d prior to conception and continuing through weaning. The top left figure shows combined labeling with anti-BrdU and anti-MBP antibodies, and the top right figure shows labeling with anti-olig2 antibodies. These images are merged in the bottom left to identify BrdU^+^/Olig2^+^ cells. Quantitative analysis of total numbers of double-positive cells reveals that developmental exposure to 100 ppb MeHg via the maternal drinking water is associated with a subtle but significant reduction in the number of O-2A/OPCs engaged in DNA synthesis, consistent with the effects of low-level MeHg exposure in vitro. Quantitative data are presented as mean percentage normalized to control animals (*n* = 3 for each group). Error bars represent ± standard error of the mean. The plus symbol indicates exposure of the cells to the indicated substance.

We found that developmental exposure of mice to MeHg at either 100 ppb or 250 ppb in the maternal drinking water was associated with clear and significant reductions in levels of PDGFRα and EGFR, but not of TrkC (Figure 11). Treatment of SJL mice with 100 or 250 ppb MeHg in the drinking water during gestation and suckling was associated with reductions in levels of PDGFRα and EGFR in the cerebellum, hippocampus, and corpus callosum when brain tissue was sampled at 7 and 21 d after birth. In contrast, levels of the NT-3 receptor TrkC were not reduced in these animals, as predicted by our in vitro analyses. It was particularly striking that exposure even to 100 ppb MeHg in the drinking water was enough to have significant effects on levels of PDGFRα and EGFR. These changes, and the lack of effect of MeHg exposure on TrkC levels, are as predicted from our in vitro analyses.

Analysis of BrdU incorporation revealed that these low levels of MeHg exposure also were associated with statistically significant reductions in the division of O-2A/OPCs in vivo. In these experiments, postnatal day 14 (P14) animals were treated as for analysis of receptor levels except that BrdU was administered 2 h before sacrifice. Sections then were analyzed with anti-BrdU antibodies to identify cells engaged in DNA synthesis and with antibodies to olig2 to identify O-2A/OPCs (as in [148]). Olig2 is a transcriptional regulator expressed in oligodendrocytes and their ancestral precursor cells (e.g., [50,149–152]. In white matter tracts of the CNS, BrdU^+^ cells that express Olig2 are considered to be O-2A/OPCs [153,154]). In our studies, greater than 90% of all BrdU^+^ cells in the corpus callosum were also Olig2^+^. When we analyzed the number of Olig2^+^/BrdU^+^ cells found in the corpus callosum of control and experimental animals (see Materials and Methods for details of analysis), we found a 20% reduction in the number both of total BrdU^+^ cells and of Olig2^+^/BrdU^+^ cells (Figure 11), an outcome in agreement with the results of our in vitro studies (Figure 2B).

## Discussion

Our studies demonstrate that chemically diverse toxicants converge on activation of a previously unrecognized pathway of cellular regulation that leads from increases in oxidative status to reductions in levels of specific RTKs. Analysis of effects of MeHg on O-2A/OPCs dividing in response to PDGF first demonstrated suppression of PDGF-induced signaling, but no reduction in NT-3–induced phosphorylation of ERK1/2. Further analysis demonstrated that MeHg exposure enhanced degradation of PDGFRα as a consequence of the sequential activation of Fyn and c-Cbl. As predicted by the hypothesis that MeHg exposure activates the redox/Fyn/c-Cbl pathway, exposure to this toxicant was also associated with reductions in levels of EGFR and c-Met (which are c-Cbl targets), but not in levels of TrkC (which is not a c-Cbl target). The redox/Fyn/c-Cbl pathway was also activated by Pb and paraquat, leading to negative modulation of RTK-mediated signaling by regulating receptor degradation and causing reductions in levels of PDGFRα, EGFR, and c-Met, but not of TrkC. Developmental exposure to MeHg was also associated with reduced levels of PDGFRα and EGFR, but not of TrkC, consistent with the hypothesis that this same regulatory pathway is activated in association with in vivo toxicant exposure.

The results of our studies are novel in a number of ways, beginning with the identification of a previously unrecognized regulatory pathway activated by chemically diverse toxicants. Although the importance of identifying general principles that apply to chemically diverse toxicants is a widely recognized goal of toxicology research, relatively few such principles have been identified. For example, although toxicants may be classified as hormonal mimetics, mutagens, carcinogens, neurotoxins, etc., relatively few mechanistic pathways have been identified on which chemically diverse substances converge.

Our present studies have identified Fyn activation as a common cellular target for the action of chemically diverse toxicants with pro-oxidant activity. Whether oxidative changes are by themselves sufficient to induce sequential activation of Fyn and c-Cbl will be a subject of continued analysis, but existing data make it difficult to imagine a compelling alternative hypothesis to explain our results. Fyn is well established as being activated when cells become more oxidized [97–100], and there is no evidence for any other unifying feature of MeHg, Pb, and paraquat that would cause Fyn activation. Activation of Fyn, and the effects of activation of the Fyn/c-Cbl pathway, were blocked by NAC (which antagonizes oxidative changes in O-2A/OPCs [59]) as effectively as by expression of Fyn-specific RNAi constructs or by pharmacological inhibition of Fyn activity. NAC protects against physiological stress in two ways, both as an anti-oxidant itself and by providing increased levels of cysteine, the rate-limiting precursor in glutathione biosynthesis (e.g., [115,116]). The ability of ProCys (which has no intrinsic anti-oxidant properties [129]) to confer similar protection as NAC suggests that it is through their enhancement of glutathione production that these two cysteine pro-drugs exert their protective effects. The relatively small effect of NAC exposure by itself on basal Fyn activity in the experimental conditions used indicates that, at least in these experiments, NAC's protective effect was more likely to be due to protection against increases in oxidative status than due to a direct suppression of Fyn activity to an extent that would neutralize the activating effects of toxicant exposure. Although increased glutathione levels theoretically could also protect against the effects of toxicants by enabling enhanced cellular export of physiological stressors (reviewed in, e.g., [155,156]), analysis with Leadmium Green AM (which can detect intracellular Pb in the nM range) revealed no apparent effect of NAC treatment on cellular levels of Pb (Figure S3). Further support for the hypothesis that transport of xenobiotics is not a likely explanation for the protective effects of NAC is also provided by ongoing studies demonstrating that TH and BMP-4 (both of which cause O-2A/OPCs to become more oxidized [59]) also cause activation of Fyn and c-Cbl, with associated reductions in PDGFRα levels (Z. Li and M. Noble, unpublished data). NAC blocks the effects of TH and BMP on differentiation, and also prevents TH- and BMP-mediated activation of Fyn and c-Cbl ([59]; Z. Li and M. Noble, unpublished data). Changes in intracellular redox state, and the predicted ability to protect with NAC, are the common features linking the activation of Fyn with MeHg, Pb, paraquat, TH, and BMP.

Although Fyn has multiple targets, it seems most likely that activation of c-Cbl provides the explanation for the effects of MeHg on PDGF-mediated signaling. Suppression of c-Cbl activity by expression of DN(70Z) c-Cbl or RNAi protected against the effects of MeHg on cell division and reductions in levels of PDGFRα. Moreover, the induction of PDGFRα ubiquitylation by MeHg, the lack of effects of MeHg on PDGFRα mRNA levels, the rescue of receptor levels by disrupting lysosomal function, and other observations all strongly indicate the importance of c-Cbl regulation in understanding the effects of toxicant exposure. The importance of Fyn in activation of c-Cbl is supported by the ability of expression of Fyn-specific RNAi, or pharmacological inhibition of Fyn activity, to protect against the effects of toxicant exposure. Because Fyn activation in O-2A/OPCs also leads to activation of Rho-GTPase, leading to inhibition of Rho kinase activity [102,157], we also examined the effects of treatment of cells with the Rho kinase inhibitor Y23762 (Figure S4). Although this agent inhibited Rho kinase activity in O-2A/OPCs, it neither protected against nor exacerbated the effects of MeHg on progenitor cell division (as determined by BrdU incorporation). Thus, although it will be of interest to examine the effects of toxicant exposure on other Fyn targets, it currently seems that Fyn-mediated activation of c-Cbl is central to understanding the effects of toxicants on O-2A/OPCs.

**Figure 12:**
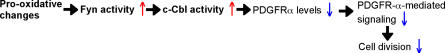
Diagrammatic Summary of the Fyn/c-Cbl Hypothesis of Toxicant Convergence The results of our studies demonstrate a regulatory network in which oxidation causes activation of Fyn. Fyn then phosphorylates c-Cbl. Activation of c-Cbl leads to ubiquitylation of agonist-activated RTKs that are c-Cbl targets, with PDGFRα used here as an example of such a receptor. Reductions in levels of receptor lead to reduced activation of downstream signaling cascades.

The discovery of sequential activation of Fyn and c-Cbl by pro-oxidants provides a new means of integrating the effects of changes in intracellular redox state with the control of the cell cycle. Although the ability of Fyn to be activated by increases in oxidative status [97–100], the functional interaction of Fyn with c-Cbl (e.g., [93–96]), and the regulation of degradation of specific RTKs by c-Cbl (e.g., see [88–90,96,106,107,134–138]) have all been subjects of study by multiple laboratories, our studies appear to provide the first integration of all of these components into a regulatory pathway of obvious relevance to the regulation of cell function by redox status. This regulatory pathway, summarized in Figure 12, offers a number of clear predictions, some of which have been tested in our present studies. Several studies on different cell types have confirmed our own finding [59] that making dividing cells more oxidized can suppress division and induce differentiation [158–160], and it will be of interest to determine the contribution of the redox/Fyn/c-Cbl pathway in these other cell systems, as well as in modulating other changes in cellular function that have been attributed to increased oxidative status (e.g., [73,161–166]).

It is particularly striking that the changes we observed were seen at environmentally relevant exposure levels for both MeHg and Pb. As many as 600,000 newborn infants in the US each year have cord blood mercury levels greater than 5.8 ppb [46] (i.e., ∼30 nM). It is reported that the blood:brain ratio for humans may be as high as 1:5 to 1:6.7 [167,168], therefore, in vivo levels in brain may be still higher than those we have studied. It is also noteworthy that levels of MeHg exposure at which selective reduction in PDGFRα expression was readily observed in vivo were 90% or more lower than exposure levels generally considered to constitute low-to-moderate exposure (e.g., [143–147]). Blood Pb levels may be of concern at levels as low as 10 μg/dl (e.g., [169–176]), which is equivalent to 0.48 μM, but which may be increased to micromolar in the brain by mechanisms relevant to Ca^2+^ transport [171]). Even given equivalence in blood:brain Pb levels, a concentration of 1 μM is equivalent to the approximately 20 μg/dl blood Pb levels known to be associated with cognitive impairment (e.g., [169–177], an exposure level of particular concern in countries where leaded gasoline is still used and in which mean blood lead levels in schoolchildren may be as high as 15 μg/dl [178].

The study of environmentally relevant levels of toxicant exposure is a great challenge, both in vitro and in vivo, and it may be that analysis of stem and progenitor cell populations will be critical in furthering such analysis. In vitro, O-2A/OPCs appear to offer a particularly useful target cell for such studies, in part due to their sensitivity to environmentally relevant exposure levels of toxicants, but also due to the ability to use clonal analysis in quantitative studies on the cumulative effects of small changes in the balance between division and differentiation [179–181]. Such studies have shown that even such potent physiological regulators as TH may only increase the probability of oligodendrocyte differentiation at each progenitor cell cycle from approximately 0.5 to 0.65 [179]. Thus, although their cumulative effects over time may be readily observable, analysis of subtle effects in acute assays may fail to identify important alterations in progenitor cell function. In addition, it will be important to extend analysis on differentiation to other precursor cell populations, as indicated by recent observations that neuronal differentiation of neuroepithelial stem cells may be compromised by MeHg exposure levels as low as 2.5 nM [182]. In vivo, the 20% reduction in number of dividing O-2A/OPCs observed in animals exposed to 100 ppb MeHg during development was of particular interest, as such relatively subtle changes might be predicted to reduce myelination in ways that require equally subtle analysis to detect functional outcomes. Analysis of conduction velocity in the auditory system may offer one such analytical tool, and the sensitivity of O-2A/OPCs to toxicant exposure may provide an explanation for the consistency with which increases in ABR latency suggestive of myelination abnormalities are associated with exposure to a variety of toxicants and physiological stressors, including MeHg [75–79], Pb [183,184]), cocaine [185,186], and carbamazepine [187].

The general importance of the signaling pathways regulated by Fyn and c-Cbl suggests that the ability of chemically diverse toxicants to converge on this pathway may be of broad relevance to the understanding of toxicant action. Such c-Cbl targets as PDGFRα, EGFR, and c-Met play critical roles in processes as diverse as cell proliferation, survival, and differentiation, cortical neurogenesis, maintenance of the subventricular zone, astrocyte development, development of cortical pyramidal dendrites, motoneuron survival and pathfinding, sympathetic neuroblast survival, and hippocampal neuron neurite outgrowth, as well as having extensive effects on development of kidney, lung, breast, and other tissues (e.g., [60,61,130,188–195]). Indeed, the range of targets of c-Cbl [92,135] offers a rich fabric of potentially critical regulatory molecules that would be affected by changes in activity of this protein, with the importance of particular proteins being dependent on the cell type and developmental stage under consideration. In addition, Fyn regulation of the Rho/ROCK signaling pathway could be of relevance in understanding toxicant-mediated alterations on such cytoskeletal functions as cell migration, neurite outgrowth, and development of dendritic morphology (e.g., [196–198]). Our studies predict that any toxicant that makes cells and/or tissues more oxidized would activate Fyn, a list that includes substances as chemically diverse as MeHg (e.g., [1–6], Pb [6–9], and organotin compounds [1,2,5,10,11]), cadmium [12,13], arsenic [12,14], ethanol [15,16], and various herbicides (e.g., paraquat [17,18], pyrethroids [19–21], and organophosphate and carbamate inhibitors of cholinesterase [22–26]).

In summary, our studies provide a new general principle and evidence of a new regulatory pathway that may be relevant to the understanding of the action of a large number of chemically diverse toxicants and other modulators of oxidative status. Because the outcomes we have identified occur at quite low toxicant exposure levels, they may provide a particularly useful unifying principle for the analysis of toxicant effects. Our present studies, combined with our previous analysis of the central importance of intracellular redox state in modulating progenitor cell function [59], lead to the prediction that any toxicant with pro-oxidant activity will exhibit these effects. Although toxicants of differing chemical structures will also have additional activities, the convergence of small increases in oxidative status on regulation of the redox/Fyn/c-Cbl pathway provides a specific means by which exposure to low levels of a wide range of chemically diverse toxicants might have similar classes of effects on development. Our findings also provide a strategy for rapid identification of such effects by any of the estimated 80,000 to 150,000 chemicals for which toxicological information is limited or nonexistent, thus enabling a preliminary identification of compounds that would need to be examined in vivo. The sensitivity of O-2A/OPCs to environmentally relevant levels of MeHg and Pb provides a great advantage over established cell lines and other such neural cells as astrocytes, for which these low exposure levels may have little effect, and the importance of understanding the effects of toxicants on progenitor cell function provides a direct link between our studies and the broad field of developmental toxicology. In addition, the ability of NAC to protect progenitor cells against the adverse effects of chemically diverse toxicants raises the possibility that this benign therapeutic agent may be of benefit in protecting children known to be at increased risk from the effects of toxicant exposure during critical developmental periods. Finally, the principles indicated by our findings appear likely to have broad applicability in understanding the regulation of cell function by alterations in redox balance, regardless of how they might be generated.

## Materials and Methods

### Cell isolation, culture, and treatment.

O-2A/OPCs were purified from corpus callosum of P7 CD rats as described previously to remove type 1 astrocytes, leptomeningeal cells, and oligodendrocytes [59,64,199]. Cells were then grown in DMEM/F12 supplemented with 1-μg/ml bovine pancreas insulin (Sigma, St. Louis, Missouri, United States), 100-μg/ml human transferrin (Sigma), 2 mM glutamine, 25-μg/ml gentamicin, 0.0286% (v/v) BSA pathocyte (ICN Biochemicals, Costa Mesa, California, United States), 0.2 μM progesterone (Sigma), 0.10 μM putrescine (Sigma), bFGF-2 (10 ng/ml; PEPRO Technologies, London, United Kingdom), and PDGF-AA (10 ng/ml; PEPRO) onto poly-l-lysine (Sigma) coated flasks or dishes. Under these conditions, O-2A/OPCs derived from the corpus callosum of P7 rats are predominantly in cell division and do not generate large numbers of oligodendrocytes during the time periods utilized in this analysis.

To generate sufficient numbers of cells for biochemical analysis, cells were expanded through one to two passages in PDGF + FGF-2 before replating in the presence of PDGF alone. When cells achieved approximately 50% confluence, MeHg, Pb, or paraquat was added to their medium at concentrations indicated in the text. Doses for the toxicants were chosen on the basis of dose-response curves to identify sublethal exposure levels (unpublished data), as a reflection of blood and brain toxicant levels of these compounds and, where applicable, on the basis of previous reports. All toxicant concentrations examined were confirmed to cause death of less than 5% of cells over the time course of the experiment.

For analysis of the effects of potential inhibitors of toxicant action, cells were exposed to the blocking compound of interest 1 h before addition of toxicant. The concentrations of inhibitors used are listed as following: 0.5 μM BIM-1 (PKC inhibitor), 0.5 μM PP1/PP2 (Src family kinase inhibitors), and 10 mM NH_4_Cl (lysosome inhibitor); and the concentrations of toxicants used are listed as following, except when mentioned specifically: MeHg (20 nM), Pb (1 μM), and paraquat (5 μM).

To examine the degradation of PDGFRα, O-2A/OPCs were treated with MeHg (20 nM) for different durations with or without cycloheximide (CHX; 1 μg/ml) added 1 h before MeHg. The cells were then collected and lysed for Western blotting. For example, in the multi-toxicant analysis of Figures 8–10, for analysis of PDGFRα, O-2A/OPCs were exposed for 24 h to MeHg (20 nM), Pb (1 μM), and paraquat (5 μM) for 24 h in the presence of 0.5 μM bisindolylmaleimide 1 (BIM-1), 0.5 μM of PP1, 1 mM NAC, or 1 mM procysteine, which had been added 1 h prior to toxicant addition. Cells were lysed for Western blot analysis using anti-PDGFRα(pY^742^) antibody. The membranes were de-probed and then re-probed with antibody against total PDGFRα and anti–β-tubulin antibody. For analysis of Fyn activity and c-Cbl phosphorylation, progenitors were exposed to MeHg (20 nM), Pb (1 μM), and paraquat (5 μM) for 3–4 h in the presence of 0.5 μM BIM1, 0.5 μM PP1,or 1 mM NAC (each of which was added 1 h before addition of toxicant).

### Cell transfection and luciferase activity assay.

Cells were deprived of PDGF-AA for 5 h before re-exposure to PDGF-AA (10 ng/ml) for 1 h for Western blot or 6 h for luciferase assays of pathway activation. Transient transfection was performed using Fugene6 (Roche, Basel, Switzerland) transfection solution according to the manufacturer's protocol. For the luciferase assay, cells seeded in 12-well plates were transfected with a reporter plasmid SRE-Luc(firefly) or NFκB-Luc(firefly) (BD-Clontech, Palo Alto, California, United States) and an internal control plasmid pRLSV40-LUC. Analyses of luciferase activity were performed according to the protocol of the Dual Luciferase Assay System (Promega, Madison, Wisconsin, United States), which uses an internal control of Renilla luciferase for quantification, and relative light units were measured using a luminometer.

### Antibodies and immunoblotting.

Anti-phosphorylated ERK monoclonal, anti-ERK monoclonal, anti-TrkC polyclonal, anti-Fyn polyclonal, anti-EGFR polyclonal, anti–c-Met polyclonal, anti–phospho-tyrosine monoclonal, and anti-PDGFRα polyclonal antibodies were obtained from Santa Cruz Biotechnology (Santa Cruz, California, United States). Anti–c-Cbl monoclonal antibody was obtained from BD PharMingen (San Diego, California, United States). Anti-phosphorylated Akt monoclonal and anti-Akt polyclonal antibodies were obtained from Cell Signaling Technology (Beverly, Massachusetts, United States). Anti-phosphorylated PDGFRα polyclonal antibody was obtained from Biosource (Carlsbad, California, United States). The cell culture samples were collected and lysed in RAPI buffer, whereas dissected tissue samples were sonicated in RAPI buffer. Samples were resolved on SDS-PAGE gels and transferred to PVDF membranes (PerkinElmer Life Science, Wellesley, Massachusetts, United States). After being blocked in 5% skim milk in PBS containing 0.1% Tween 20, membranes were incubated with a primary antibody, followed by incubation with an HRP-conjugated secondary antibody (Santa Cruz Biotechnology). Membranes were visualized using Western Blotting Luminol Reagent (Santa Cruz Biotechnology). All analyses of signaling pathway components were conducted in the presence of ligand for the receptor pathway under analysis (either PDGF-AA for PDGFRα, NT-3 or TrkC, HGF for c-Met, or EGF for EGFR).

### In vitro BrdU incorporation assay.

Cell proliferation was assessed by bromodeoxyuridine (BrdU) incorporation and by using the mouse anti-BrdU mAb IgG_1_ (1:100; Sigma) to label dividing cells. Stained cells on coverslips were rinsed two times in 1× PBS, counterstained with 4′6-diamidino-2-phenylindole (DAPI; Molecular Probes, Eugene, Oregon, United States) and mounted on glass slides with Fluoromount (Molecular Probes). Staining against surface proteins was performed on cultures of living cells or on cells fixed with 2% paraformaldehyde. Staining with intracellular antibodies was performed by permeabilizing cells with ice-cold methanol for 4 min or by using 0.5% Triton for 15 min on 2% paraformaldehyde–fixed cells. Antibody binding was detected with appropriate fluorescent dye–conjugated secondary antibodies at 10 μg/ml (Southern Biotech, Birmingham, Alabama, United States) or Alexa Fluor–coupled antibodies at a concentration of 1 μg/ml (Molecular Probes), applied for 20 min. Anti-BrdU monoclonal antibody was obtained from Sigma.

### Intracellular reactive oxygen species measurement and analysis of Pb uptake.

Cells were plated in 96-well microplates and grown to about 60% confluence. Prior to treatment, cells were washed twice with Hank's buffered saline solution (HBSS), loaded with 20 μM H2DCFDA (in HBSS 100 μl/well), and incubated at 37 °C for 30 min. Cells were then washed once with HBSS and growth medium to remove free probe. Then, fresh growth medium was added and a baseline fluorescence reading was taken prior to treatment. For NAC pre-treatment, NAC was added into media 1 h before further addition of MeHg, and both compounds remained in the medium during the incubation period with H2DCFDA. Fluorescence was measured in a Wallac 1420 Victor^2^ multilabel counter (PerkinElmer) using excitation and emission wavelengths of 485 nm and 535 nm, respectively, at different time courses as indicated in the figures. Results are presented as the value change from baseline by the formula (Ft_exp_ − Ft_base_)/Ft_base_ normalized with the control group, where Ft_exp_ = fluorescence at any given time during the experiment in a give well and Ft_base_ = baseline fluorescence of the same well.

We further determined whether pre-treatment with NAC altered levels of intracellular Pb by analysis with the Leadmium Green AM dye (Molecular Probes), according to the manufacturer's instructions. In five separate experiments, we found no significant difference between O-2A/OPCs treated with 1 μM Pb versus [Pb + NAC] (unpaired *t*-test), and the values for both Pb-treated samples were several-fold higher than control values. All of these data strongly support the hypothesis that the major effect of NAC is to antagonize cellular oxidation.

### Immunoprecipitation assay.

For the co-immunoprecipitation assay, anti-c-Cbl monoclonal antibody (BD PharMingen) or anti-PDGFRα polyclonal antibody (Santa Cruz Biotechnology) was added to the pre-cleared cell lysates (250 μg of total protein), and the mixtures were gently rocked for 2 h at 4 °C. A total of 30 μl of protein A/G agarose was then added to the mixture followed by rotating at 4 °C overnight. The protein A/G agarose was then spun down and washed thoroughly three times. The precipitates were resolved on an 8% SDS-PAGE gel and then were subjected to Western blot analysis using an anti-p-Tyr (for c-Cbl phosphorylation assay) or ubiquitin (for PDGFR ubiquitination assay) antibody (Santa Cruz Biotechnology).

### Fyn kinase assay.

Fyn kinase activity was quantified using the Universal Tyrosine Kinase Assay Kit (Takara, Madison, Wisconsin, United States). O-2A/OPCs exposed to different treatments were solubilized with an equal volume of the extraction buffer provided with the kit for 15 min, and the resulting lysates were centrifuged at 13,000 × *g* for 15 min at 4 °C; 250 μg of total cell lysates were immunoprecipitated with anti-Fyn antibody (Santa Cruz Biotechnology). Following immunoprecipitation, Fyn immune complexes were washed four times with extraction buffer, and then Fyn kinase activities of each sample were assayed using the kit according to the manufacturer's instructions.

### Rho kinase assay.

Rho kinase activity was quantified using the CycLex Rho-Kinase Assay kit (MBL International, Woburn, Massachusetts, United States) as described. Cells were lysed and about 500 μg of total cell lysates were immunoprecipitated with anti-ROCK1 antibody (Sigma), and the precipitates were re-suspended with kinase reaction buffer provided in the kit. Rho kinase activities of each sample were assayed using the kit according to the manufacturer's instructions.

### DNA vector-based RNA interference.

siRNA target sites were selected by scanning the cDNA sequence for AA dinucleotides via siRNA target finder (Ambion, Austin, Texas, United States). Those 19-nucleotide segments that start with G immediately downstream of AA were recorded and then analyzed by BLAST search to eliminate any sequences with significant similarity to other genes. The siRNA inserts, containing selected 19-nucleotide coding sequences followed by a 9-nucleotide spacer and an inverted repeat of the coding sequences plus 6 Ts, were made to double-stranded DNAs with ApaI and EcoRI sites by primer extension, and then subcloned into plasmid pMSCV/U6 at the ApaI/EcoRI site. The corresponding oligonucleotides for the Fyn and c-Cbl RNAi's are listed in Table 1. Several nonfunctional siRNAs, which contain the scrambled nucleotide substitutions at the 19-nucleotide targeting sequence of the corresponding RNAi sequence, were constructed as negative controls. All of these plasmids were confirmed by complete sequencing.

**Table 1:**
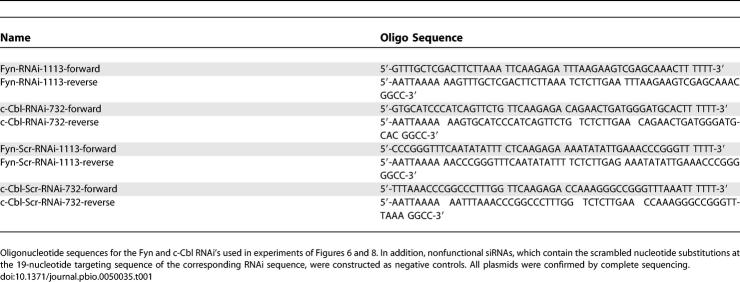
Oligonucleotide Sequences for siRNAs

### Viral packaging, cell infection, and selection.

pJEN/neo-HA-70z-c-Cbl plasmids were generously provided by Dr. Wallace Langdon. The pBabe(puro)-HA-70z-c-Cbl plasmids were constructed by transferring the BamH1-digested HA-70z-c-Cbl from pJEN/neo-HA-70z-c-Cbl into the BamH1 digested pBabe(puro) vector. The pBabe(puro)-HA-70z-c-Cbl, pMSCV/U6-Fyn-RNAi, pMCV/U6-c-Cbl-RNAi, and the corresponding scrambled RNAi plasmids and the empty plasmids were transfected into Pheonix Ampho cells by Fugene6 (Roche) transfection solution according to the manufacturer's protocol. Twenty-four hours after transfection, medium was changed to DMEM/F12(SATO, but with no TH) supplemented with 10-ng/ml PDGF-AA and bFGF. Virus supernatant was collected 48 h post-transfection, filtered through 0.45-μm filter to remove non-adherent cells and cellular debris, frozen in small aliquots on dry ice, and stored at −80 °C. Twenty-four hours prior to infection, O-2AOPCs were seeded. The following day, the culture medium was aspirated and replaced with virus supernatant diluted 1:1 in the O-2A growth media. Medium was then changed into O-2A/OPC growth medium after 8 h or overnight. Twenty-four hours after infection, the cells were collected by trypsinization and reseeded in the selective medium (growth medium + 200-ng/ml puromycin). By the next day, all noninfected cells were floating and presumably dead or dying. The infected cells were allowed to proliferate for 2 d, and then collected and re-seeded for the following experiments.

### RNA isolation and real-time RT-PCR.

Total RNA was isolated using TRIZOL reagent (Invitrogen, Carlsbad, California, United States) according to the manufacturer's protocol. A total of 1 μg of RNA was subjected to reverse transcription using Superscript II (Invitrogen). The reactions were incubated at 42 °C for 50 min. The FAM-labeled probe mixes for rat PDGFRα and Fyn, and the VIC-labeled GAPDH probe mix were purchased from Applied Biosystems (Foster City, California, United States). For multiplex real-time PCR, reactions each containing 5 μl of 10-fold–diluted reverse transcription product, 1 μl of interest gene probe mix, 1 μl of GAPDH probe mix, and 10 μl of TaqMan Universal PCR Master Mix were performed on an iCycler iQ multicolor real-time PCR system (Bio-Rad, Hercules, California, United States) and cycling condition was 50 °C for 2 min and 95 °C for 10 min, followed by 40 cycles of 95 °C for 15 sec and 60 °C for 1 min. Each sample was run in triplicate. Data were analyzed by iCycler iQ software (Bio-Rad).

### Clonal analysis.

O-2A/OPCs purified from P7 rat optic nerve were plated in poly-L-lysine–coated 25-cm^2^ flasks at clonal density with DMEM medium in the presence of 10-ng/ml PDGF as previously described [59,64,199]. After 24-h recovery, cells were treated with different toxicants, each for 3 d, until visual inspection and immunostaining was performed. NAC was added 1 h before exposure to other toxicants for NAC pretreatment, and NAC co-exists throughout the culture period. The numbers of O-2A/OPCs and oligodendrocytes in each clone were determined by counting under fluorescent microscope. The three-dimensional graph shows the number of clones containing O-2A/OPC cells and oligodendrocytes. Experiments were performed in triplicate in at least two independent experiments.

### Animal treatment.

Six-week-old female SJL mice were treated with MeHg in their drinking water at a concentration of 100 or 250 ppb for 30–60 d prior to mating, and then throughout pregnancy and gestation. This is a level of treatment that is 75%–90% below levels generally considered to be low to moderate and is below levels that have been associated with gross defects in adult or developing animals (e.g., [143–147]).

The exposure levels used in our studies were first determined as candidate exposures from the results of two different previous studies on the relationship between MeHg exposure and levels of toxicant in the brain. Studies by Weiss and colleagues [143] demonstrated that mice exposed to MeHg in their drinking water for up to 14 mo have brain mercury levels roughly equivalent to that in the water. In these studies, mice exposed to MeHg in their drinking water from conception at a concentration of one part per million (ppm) had brain levels of MeHg of 1.20 mg/kg (i.e., ppm) at 14 mo of age, whereas those exposed to MeHg at a concentration of 3 ppm had brain levels of 3.66 mg/kg at this age. It has also been shown, however, that mercury levels in the brain of pre-weanling animals exposed to MeHg via the mother's drinking water throughout gestation and suckling drop rapidly to one-fifth of the levels found at birth, presumably due to reduced MeHg transfer in milk [200]. As an estimated 300,000 to 600,000 infants in the US have blood cord mercury levels of 5.8 μg/l or more [46], and because the human brain concentrates MeHg 5- to 6.7-fold over the concentration occurring in the bloodstream, our goal was to achieve postnatal brain mercury levels of 30 ppb (i.e., ng/g) or less.

In practice, we found that exposure of female mice to MeHg in their drinking water at a concentration of 250 ppb prior to conception, and maintenance of this exposure during suckling, was associated with brain mercury levels in the offspring (examined at P14) of 50 ng/g, a fall that was precisely in agreement with predictions based on prior studies on the fall of mercury levels occurring during this period in suckling mice [200]. In offspring of dams exposed to MeHg at a concentration of 100 ppb in the drinking water, brain mercury was below the levels of detection of the Mercury Analytical Laboratory of the University of Rochester Medical Center. The exposure levels of 100 and 250 ppb are 75%–90% below what has otherwise been considered to be low-dose exposure in mice.

### Tissue preparation.

At the time of sacrifice, mice were anesthetized using Avertin (tribromoethanol, 250 mg/kg, 1.2% solution; Sigma) and were perfused transcardially with 4% paraformaldehyde in phosphate buffer (pH 7.4) following the removal of the blood by saline solution washing. The brains were removed and stored in 4% paraformaldehyde for 1 d, and then changed to 25% sucrose in 0.1 M phosphate buffer. Brains were cut coronally as 40-μm sections with a sliding microtome (SM/2000R; Leica, Heidelberg, Germany) and stored at −20 °C in cryoprotectant solution (glycerol, ethylene glycol, and 0.1 M phosphate buffer[ pH 7.4], 3:3:4 by volume). All animal experiments were conducted in accordance with National Institutes of Health guidelines for the humane use of animals.

### In vivo BrdU incorporation assay, BrdU labeling, and olig2 co-labeling for BrdU detection.

To analyze DNA synthesis in vivo, mice were injected with a single dose of 5-BrdU (50 mg/kg body weight), dissolved in 0.9% NaCl, filtered (0.2 μm), and applied intraperitoneally 2 h prior to perfusion. After removal and sectioning of brains, 40-μm free-floating sections were incubated for 2 h in 50% formamide/2× SSC (0.3 M NaCl and 0.03 M sodium citrate) at 65 °C, rinsed twice for 5 min each in 2× SSC, incubated for 30 min in 2N HCl at 37 °C, and rinsed for 10 min in 0.1 M boric acid (pH 8.5) at room temperature. Several rinses in TBS were followed by incubation in TBS/0.1% Triton X-100/3% donkey serum (TBS-plus) for 30 min. Sections were then incubated with monoclonal rat anti-BrdU antibody (1:2,500; Harlan Sera-Lab, Loughborough, United Kingdom) and polyclonal rabbit anti-Olig2 (a generous gift from Dr. David H. Rowitch) in TBS-plus for 48 h at 4 °C. Sections were rinsed several times in TBS-plus and incubated for 1 h with donkey anti-rat FITC and donkey anti-rabbit TRITC (Jackson ImmunoResearch Laboratories, West Grove, Pennsylvania, United States). After several washes in TBS, sections were mounted on gelatin-coated glass slides using Fluoromount-G mounting solution (Southern Biotech).

Quantification of BrdU^+^ cells was accomplished with unbiased counting methods by confocal microscopy. BrdU immunoreactive nuclei were counted in one focal plane to avoid oversampling. In corpus callosum, BrdU^+^ cells were counted in every sixth section (40 μm) from a coronal series between interaural AP + 5.2 mm and AP + 3.0 mm in the entire extension of the rostral and medial part of the corpus callosum. Quantitative data are presented as mean percentage normalized to control animals. Error bars represent ± the standard error of the mean.

### Images, data processing, and statistics.

Digital images were captured using a confocal laser scanning microscope (Leica TCS SP2). Photomicrographs were processed on a Macintosh G4 and assembled with Adobe Photoshop 7.0 (Adobe Systems, Mountain View, California, United States). Unpaired, two-tailed Student *t*-test was used for statistical analysis.

## Supporting Information

Figure S1 MeHg-Induced Reductions in Levels of PDGFRα Are Not Dependent upon Changes in Transcription of PDGFRα mRNA and Exceed Normal Levels of Receptor Loss in Untreated Cells Cells were grown as for Figure 5.
(A) Despite the reduction in levels of PDGFRα associated with MeHg exposure, this toxicant had no apparent effect on levels of PDGFRα mRNA, as detected by quantitative PCR analysis.
(B) Inhibition of protein synthesis with cycloheximide (CHX) was associated with reductions in levels of PDGFRα, but the level of receptor loss occurring when MeHg was also present was markedly more severe. All results are as predicted by the hypothesis that the primary cause of reduced levels of PDGFRα result from activation of c-Cbl, leading to enhanced degradation of activated PDGFR. All experiments were repeated at least three times.
(243 KB TIF)

Figure S2 The Effects of MeHg, Pb, and Paraquat Were Not Overridden by BIM-1, a Broad-Spectrum PKC Inhibitor O-2A/OPCs were exposed to MeHg (20 nM), Pb (1 μM), or paraquat (5 μM) for 24 h with the presence of 0.5 μM of bisindolylmaleimide 1 (BIM-1) or 0.5 μM of PP1.
(A) Toxicant-induced increases in Fyn activity were prevented by co-exposure to PP1, but not to BIM-1.
(B) BIM-1 co-exposure did not protect against Pb- or paraquat-induced increases in c-Cbl phosphorylation.
(C) Toxicant-induced reductions in levels of PDGFRα were prevented by co-exposure of cells to PP1, but not by co-exposure to BIM-1. All experiments were repeated at least three times, and all numerical values represent means ± standard deviation (SD) for triplicate data points. *, *p* < 0.05; **, *p* < 0.01.
(391 KB TIF)

Figure S3 Analysis with Leadmium Green AM Demonstrates That NAC Did Not Reduce the Levels of Pb in O-2A/OPCs Cells were incubated in the presence of 1 μM Pb, 1 mM NAC, both together, or neither as for Figure 8. Over five separate experiments, we found no significant difference between O-2A/OPCs treated with 1 μM Pb versus [Pb + NAC] (unpaired *t*-test), and the values for both Pb-treated samples were several-fold higher than control values.
(43 KB PDF)

Figure S4 Inhibition of Rho Kinase Does Not Alter MeHg-Associated Suppression of BrdU Incorporation Cells were treated as for analysis of effects of MeHg on PDGF-mediated signaling.
(A) The addition of Rho kinase inhibitor Y27632 did not alter the effects of MeHg on O-2A/OPC proliferation.
(B) The concentration of Y27632 used successfully inhibited Rho kinase activity.
(85 KB PDF)

## Abbreviations

BMP: bone morphogenetic protein
CNS: central nervous system
EGF: epidermal growth factor
EGFR: epidermal growth factor receptor
ERK: extracellular signal-regulated kinase
FGF-2: fibroblast growth factor-2
MeHg: methylmercury
NT-3: neurotrophin-3
O-2A/OPC: oligodendrocyte-type-2 astrocyte progenitor/oligodendrocyte precursor cell
PDGF: platelet-derived growth factor
PDGFRα: platelet-derived growth factor receptor α
PKC: protein kinase C
ppb: parts per billion
RNAi: inhibitory RNA
RTK: receptor tyrosine kinases
SD: standard deviation
siRNA: small interfering RNA
SRE: serum response element
TH: thyroid hormone
